# Selective regulation of a defined subset of inflammatory and immunoregulatory genes by an NF-κB p50–IκBζ pathway

**DOI:** 10.1101/gad.351630.124

**Published:** 2024-06-01

**Authors:** Allison E. Daly, George Yeh, Sofia Soltero, Stephen T. Smale

**Affiliations:** 1 Department of Microbiology, Immunology, and Molecular Genetics, University of California, Los Angeles, Los Angeles, California 90095, USA;; 2 Molecular Biology Institute, University of California, Los Angeles, Los Angeles, California 90095, USA;; 3 Howard Hughes Medical Institute, University of California, Los Angeles, Los Angeles, California 90095, USA

**Keywords:** macrophages, inflammation, innate immunity, transcription, NF-κB, IκBζ

## Abstract

The five NF-κB family members and three nuclear IκB proteins play important biological roles, but the mechanisms by which distinct members of these protein families contribute to selective gene transcription remain poorly understood, especially at a genome-wide scale. Using nascent transcript RNA-seq, we observed considerable overlap between p50-dependent and IκBζ-dependent genes in Toll-like receptor 4 (TLR4)-activated macrophages. Key immunoregulatory genes, including *Il6*, *Il1b*, *Nos2*, *Lcn2,* and *Batf,* are among the p50–IκBζ-codependent genes. IκBζ-bound genomic sites are occupied at earlier time points by NF-κB dimers. However, p50–IκBζ codependence does not coincide with preferential binding of either p50 or IκBζ, as RelA co-occupies hundreds of genomic sites with the two proteins. A common feature of p50–IκBζ-codependent genes is a nearby p50/RelA/IκBζ-cobound site exhibiting p50-dependent binding of both RelA and IκBζ. This and other results suggest that IκBζ acts in concert with RelA:p50 heterodimers. Notably, p50–IκBζ-codependent genes comprise a high percentage of genes exhibiting the greatest differential expression between TLR4-stimulated and tumor necrosis factor receptor (TNFR)-stimulated macrophages. Thus, our genome-centric analysis reveals a defined p50–IκBζ pathway that selectively activates a set of key immunoregulatory genes and serves as an important contributor to differential TNFR and TLR4 responses.

## Abstract (precis)

In this study, Daly et al. report a p50- and IκBζ-dependent coregulatory axis that controls the expression of a subset of codependent inflammatory genes in activated macrophages. IκBζ binds NF-κB-primed accessible chromatin and, in concert with RelA:p50 heterodimers, promotes differential, immunoregulatory transcriptional programs in response to TLR4 and TNFR stimulation.

Inflammation is activated by diverse physiological stimuli, including microbial agents, cytokines, and numerous environmental insults. A critical component of an inflammatory response is the transcriptional activation of proinflammatory genes that are tailored to the stimulus to defend the host and restore homeostasis. The transcriptional response is dictated by the sensors of the stimulus, the signaling pathways and transcription factors induced by the sensors, and the poised and stimulus-responsive chromatin state of the genome (Heinz et al. 2015; Monticelli and Natoli 2017; Sheu and Hoffmann 2022).

Differential responses to the microbial product lipid A and the cytokine tumor necrosis factor (TNF) have long served as a model for understanding the selectivity of proinflammatory gene induction (Covert et al. 2005; Werner et al. 2005). These stimuli, sensed by Toll-like receptor 4 (TLR4) and the TNF receptors (TNFRs), respectively, induce common transcription factors, including NF-κB, AP-1, and serum response factor (SRF) (Takeuchi and Akira 2010; Hayden and Ghosh 2012; Brenner et al. 2015). However, despite extensive overlap, the transcriptional responses to TLR4 and TNFRs exhibit important differences. Notably, the type 1 interferon (IFN) response and several key effector molecules are selectively activated by TLR4 signaling. A prominent contributor to this differential response is TLR4's ability to activate the TRIF signaling pathway and its downstream IRF3 transcription factor (Yamamoto et al. 2002; Fitzgerald et al. 2003; Oshiumi et al. 2003). The TRIF pathway also prolongs NF-κB activation, allowing activation kinetics to serve as a contributor to the differential responses (Covert et al. 2005; Werner et al. 2005; Cheng et al. 2021).

Although many studies of NF-κB have focused on the abundant heterodimer composed of the NF-κB RelA and p50 subunits, the NF-κB family consists of five members (RelA, c-Rel, RelB, p50, and p52), each of which contains a conserved Rel homology region (RHR) that supports DNA binding and dimerization (Hayden and Ghosh 2012). The five subunits assemble into 15 possible homodimeric and heterodimeric species, with the RelA, c-Rel, and RelB subunits containing transactivation domains. Most NF-κB dimers are induced by post-translational mechanisms in response to inflammatory stimuli (Hayden and Ghosh 2012). Phenotypic studies of mice lacking the genes encoding NF-κB subunits have revealed distinct immunological defects (Hayden and Ghosh 2012). However, much remains to be learned about dimer-specific functions and the mechanisms of dimer-specific regulation.

The p50 protein, encoded by the *Nfkb1* gene, lacks a transactivation domain. However, it has been proposed to regulate transcription via diverse mechanisms as a subunit of multiple dimeric species. First, the abundant RelA:p50 and c-Rel:p50 heterodimers support transcriptional activation of numerous genes, with the RelA and c-Rel subunits providing the transactivation domain (Hayden and Ghosh 2012). In addition, p50 homodimers are thought to serve as repressors due to their absence of a transactivation domain and potential to compete for binding with other NF-κB dimers (Cheng et al. 2011; Zhao et al. 2012). Interactions between p50 homodimers and histone deacetylases also contribute to repression (Zhong et al. 2002; Elsharkawy et al. 2010). Finally, p50 interacts with nuclear inhibitor of NF-κB (IκB) proteins, which serve as coactivators or corepressors (Hayden and Ghosh 2012).

The mouse and human genomes encode eight IκB proteins, three of which—IκBα, IκBβ, and IκBε—sequester NF-κB dimers in the cytoplasm prior to cell stimulation (Hayden and Ghosh 2012). Two other IκB-like proteins are components of the precursors to the NF-κB p50 and p52 proteins. The remaining three IκB proteins—IκBζ (*Nfkbiz*), IκBNS (IκBδ and *Nfkbid*), and Bcl3 (*Bcl3*)—are found in the nucleus, and each interacts with the NF-κB p50 protein (Hayden and Ghosh 2012; Chiba et al. 2013; Schuster et al. 2013; Annemann et al. 2016; Willems et al. 2016). IκBζ primarily serves as a transcriptional coactivator, IκBNS is primarily a corepressor, and Bcl3 has been proposed to contribute to both activation and repression.

Although much has been learned, the regulatory logic through which different NF-κB dimers and nuclear IκBs contribute to selective gene regulation remains poorly understood. This knowledge has been difficult to achieve for multiple reasons, including redundancy between dimeric species, similarity of binding motifs, and the complexity of the mutant phenotypes.

To gain further insight, we first performed nascent transcript RNA-seq with bone marrow-derived macrophages (BMDMs) from wild-type (WT) and *Nfkb1*^−/−^ mice. This analysis revealed a small number of key inflammatory genes that exhibit strong p50 dependence, a large percentage of which also exhibit dependence on IκBζ. ChIP-seq analyses reveal extensive co-occupancy of thousands of genomic sites by p50, RelA, and IκBζ. A strong enrichment of p50–IκBζ-codependent genes occurs near a small subset of genomic sites where both IκBζ binding and RelA binding are dependent on p50. These findings and others suggest that IκBζ interacts functionally with RelA:p50 heterodimers. In addition, kinetic studies demonstrate that IκBζ binds sites assembled into open chromatin that are prebound by NF-κB dimers, with IκBζ binding correlating with the acquisition of the histone H3K27ac mark representative of a transcriptionally active state. Importantly, we found that both IκBζ mRNA and the set of p50–IκBζ-codependent genes are among the genes that exhibit the greatest differential expression in response to TLR4 versus TNFR signaling. Therefore, the weak expression of IκBζ in response to TNFR signaling is likely to represent a third major contributor to differential TLR4 and TNFR responses.

## Results

### NF-κB p50-dependent transcription of TLR4-induced genes

To uncover the logic through which distinct NF-κB family members coordinate selective responses, we first performed RNA-seq with nascent chromatin-associated transcripts from wild-type (WT) and *Nfkb1*^−/−^ BMDMs stimulated with lipid A for 0, 0.5, 1, 2, and 6 h. We examined nascent transcripts (Bhatt et al. 2012) to focus on transcriptional differences rather than possible differences in mRNA stability.

**Figure 1.**
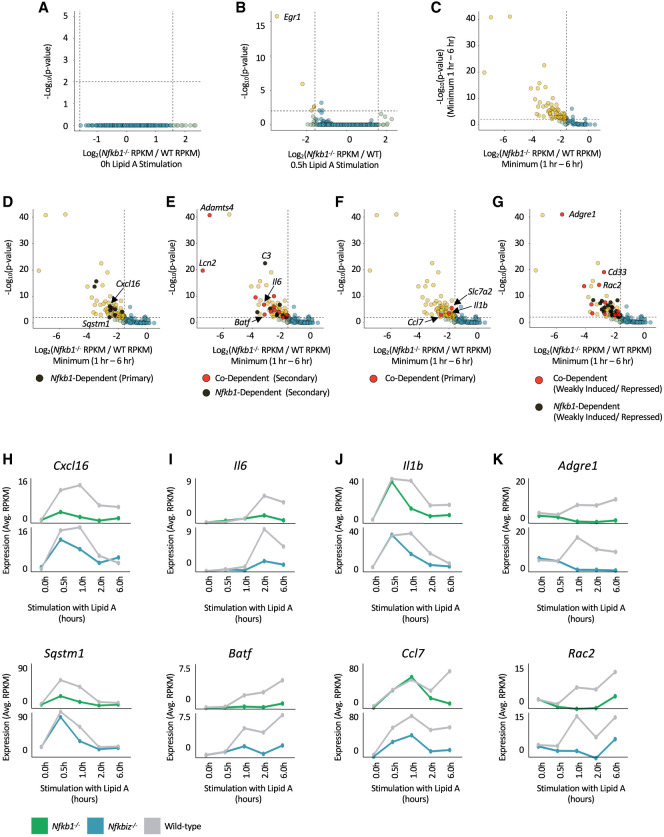
Nascent RNA-seq analysis of *Nfkb1*^−/−^ and *Nfkbiz*^−/−^ BMDMs. (*A*) A volcano plot comparing unstimulated *Nfkb1*^−/−^ and WT BMDMs is shown, with dots representing all genes considered to be expressed (RPKM > 3) in any of the samples (0, 0.5, 1, 2, or 6 h). No genes were either positively or negatively regulated to a statistically significant extent prior to stimulation. (*B*) A volcano plot comparing *Nfkb1*^−/−^ and WT BMDMs stimulated with lipid A for 0.5 h is shown. Genes with greater dependence and significance than our thresholds (dashed lines) for RPKM ratio (<33%) and *P*-value (<0.01) are in gold. (*C*) A volcano plot comparing *Nfkb1*^−/−^ and WT BMDMs stimulated with lipid A for 1, 2, and 6 h is shown. The values (ratio and *P*-value) used for each gene were derived from the time point showing the minimum *Nfkb1*^−/−^:WT RPKM ratio (WT RPKM must be >3). Genes with greater dependence and significance than our thresholds (dashed lines) for RPKM ratio (<33%) and *P*-value (<0.01) are in gold. (*D*–*G*) The volcano plot from *C* is shown with specific gene categories highlighted. (*D*) *Nfkb1*-dependent primary response genes are highlighted in black. (*E*) *Nfkb1*-dependent secondary response genes are shown in black, and *Nfkb1/Nfkbiz*-codependent secondary response genes are in red. (*F*) *Nfkb1/Nfkbiz*-codependent primary genes are highlighted in red. (*G*) *Nfkb1-*dependent (black) and *Nfkb1/Nfkbiz*-codependent (red) genes that are only weakly induced (induction from onefold to fivefold) or repressed by lipid A in WT BMDMs are highlighted; these genes do not reach our thresholds for classification as primary or secondary response genes. (*H*–*K*) Line graphs displaying nascent transcript kinetics across the time course for representative *Nfkb1*-dependent primary response genes (*H*), *Nfkb1/Nfkbiz*-codependent secondary response genes (*I*), *Nfkb1/Nfkbiz*-codependent primary response genes that display statistical dependence only at late time points after an initial primary response activation (*J*), and *Nfkb1/Nfkbiz*-codependent genes that are only weakly induced or repressed in WT BMDMs (*K*). Transcript levels are shown for *Nfkb1*^−/−^ and *Nfkbiz*^−/−^ BMDMs, along with WT BMDMs analyzed in parallel with the mutant cells. Transcript levels represent averages of two biological replicates.

In unstimulated BMDMs, the absence of p50 had little effect on nascent transcript levels (Fig. 1A). Although p50 homodimers are thought to contribute to transcriptional repression (see above), the absence of p50 in unstimulated BMDMs was insufficient to significantly alter the transcription of any genes. Interestingly, 0.5 h after stimulation, only four genes exhibit significant (*P*adj < 0.01) and relatively strong (<33% relative to WT) dependence on p50 (Fig. 1B). *Egr1* stands out, as it was induced 129-fold in WT BMDMs but only eightfold in the mutant BMDMs. In contrast, transcript levels for the other three genes (*Hmga1b*, *Slc11a2*, and *Lfng*) were only minimally induced or repressed in WT BMDMs (1.4–2.5-fold).

*Egr1*’s uniquely strong transcript reduction in *Nfkb1*^−/−^ BMDMs at the 0.5 h time point is likely due to its dependence on the mitogen-activated protein kinases (MAPKs) ERK1 and ERK2, which activate serum response factor (SRF)-associated ternary complex factors (TCFs) that are critical for *Egr1* transcription (Hill and Treisman 1995). ERK1/2 activation is dependent on the MAPK kinase kinase Tpl2, whose activity is strongly deficient in *Nfkb1*^−/−^ macrophages. Tpl2 stability requires association with the ankyrin repeat-containing p105 precursor protein encoded by the *Nfkb1* gene (Waterfield et al. 2003; Beinke et al. 2004; Gantke et al. 2011). RNA-seq analyses with lipid A-stimulated *Map3k8*^*D270A/D270A*^ (kinase-inactive Tpl2 mutant) BMDMs (Blair et al. 2022) and with lipid A-stimulated WT BMDMs in the presence of an ERK1/2 inhibitor (Supplemental Fig. S1) demonstrated that *Egr1* is the most strongly impacted gene in both settings. Thus, *Egr1* deficiency in *Nfkb1*^−/−^ BMDMs is likely due to the absence of Tpl2.

At 1, 2, and 6 h after stimulation, only 65 genes (WT RPKM > 3) exhibit strongly diminished transcript levels at one or more time points in the *Nfkb1*^−/−^ BMDMs (<33% relative to WT; *P*-value < 0.01) (Fig. 1C; also see heat maps in Supplemental Figs. S2, S3). This gene set included only nine genes classified previously (Tong et al. 2016) or in this study (Supplemental Figs. S2, S3) as primary response genes: *Egr1*, *Nfkb2*, *Kdm6b*, *Cxcl16*, *Sqstm1*, *Flnb*, *Gadd45b*, *Tnip1*, and *Gpr132* (Fig. 1D, black dots; Supplemental Fig. S2). Figure 1H shows kinetic profiles for *Cxcl16* and S*qstm1* as examples. The p50-dependent gene set also included 20 genes with secondary response characteristics (i.e., CHX-sensitive induction) (Tong et al. 2016), including *Il6*, *Nos2*, *Il4i1*, *C3*, *Stat5a*, *Adora2a*, *Lcn2*, *Adamts4*, *Batf*, and others (codependence discussed below) (Fig. 1E, black and red dots; Supplemental Figs. S2, S3). Figure 1I shows kinetic profiles for *Il6* and *Batf* as examples. Notably, none of our previously defined IFNAR-dependent secondary response genes (Tong et al. 2016) exhibit p50 dependence (data not shown), demonstrating that p50 is not necessary for the type I IFN response.

Three additional primary response genes (*Il1b*, *Ccl7*, and *Slc7a2*) exhibit p50 dependence but only at later time points (Fig. 1F, red dots; Supplemental Fig. S2). Transcript profiles for two of these genes, *Il1b* and *Ccl7* (Fig. 1J), display their rapid p50-independent induction at early time points, followed by p50 dependence at later time points. Thus, although p50 is not required for the initial activation of these genes (in contrast to the early p50 dependence of other primary response genes) (Fig. 1H), their sustained transcription is strongly influenced by p50. The remaining 35 genes that meet our criteria for p50 dependence are induced only weakly or are repressed following stimulation, with p50 dependence generally observed at the 1 h time point or later (Fig. 1G,K). In general, transcript levels for these genes are higher in WT cells than in *Nfkb1*^−/−^ cells at late time points, demonstrating a role for p50 in their sustained transcription.

To summarize, although p50 can contribute to TLR4-induced transcription via multiple mechanisms, p50-dependent transcription is most pronounced at a small number of inducible genes. Because this group includes several key immunoregulatory genes, further examination of the underlying mechanism is warranted.

### Initial analysis of nuclear IκBs

**Figure 2.**
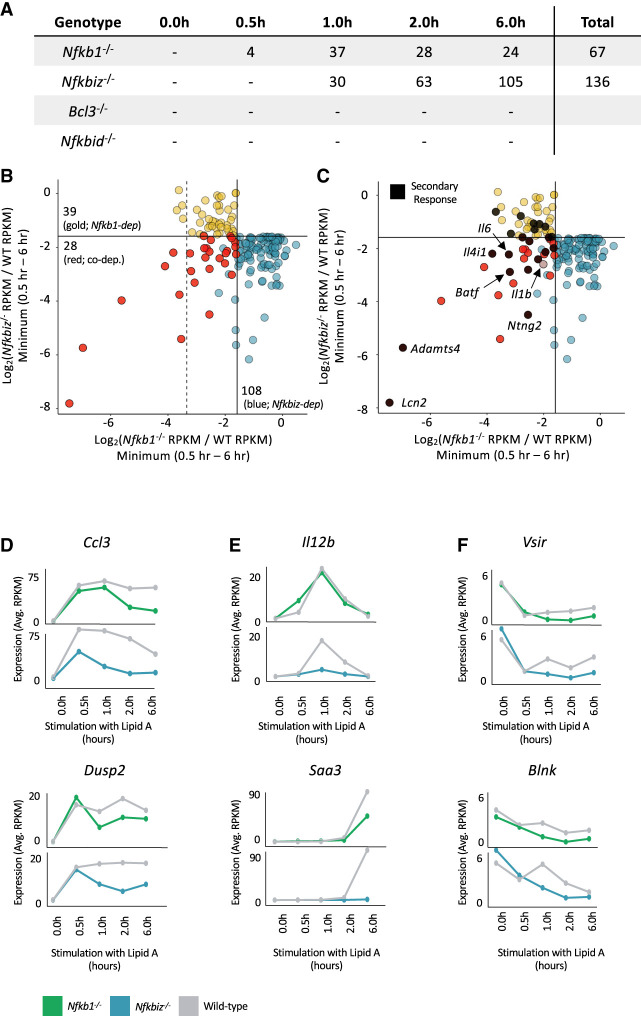
Nascent RNA-seq analysis of *Nfkbiz*^−/−^, *Bcl3*^−/−^, and *Nfkbid*^−/−^ BMDMs. (*A*) The total number of *Nfkb1*^−/−^-, *Nfkbiz*^−/−^-, *Bcl3*^−/−^-, or *Nfkbid*^−/−^-dependent genes (<33% relative to WT; *P*-value < 0.01; RPKM > 3) at each lipid A stimulation time point is shown. (*B*) The scatter plot reveals genes exhibiting *Nfkb1* and/or *Nfkbiz* codependence. Log_2_ ratios of *Nfkb1*^−/−^ RPKM:WT RPKM (*x*-axis) and *Nfkbiz*^−/−^ RPKM:WT RPKM (*y*-axis) were plotted. The values used for each gene were derived from the time point (0.5, 1, 2, or 6 h) resulting in the smallest RPKM ratio (WT RPKM must be >3). Solid lines represent an RPKM ratio of 0.33. Genes showing *Nfkb1/Nfkbiz* codependence, *Nfkb1* dependence, or *Nfkbiz* dependence are in red, gold, and blue, respectively. To be considered dependent or codependent, a gene must be below both our ratio (0.33) and *P*-value (0.01) thresholds. Thus, genes colored blue in the *bottom left* quadrant are not colored red because their *P*-values are not below the *P*-value threshold for *Nfkb1* dependence. The dashed line corresponds to an *Nfkb1*^−/−^ RPKM:WT RPKM ratio of 0.1 to highlight the fact that seven of 11 genes with the greatest *Nfkb1* dependence display *Nfkb1/Nfkbiz* codependence. (*C*) The scatter plot from *B* is reproduced with *Nfkb1*-dependent secondary response genes in black. *Il1b*, a primary response gene with *Nfkb1/Nfkbiz* codependence at late time points, is also noted. (*D*–*F*) Line graphs displaying nascent transcript kinetics across the time course for representative *Nfkbiz*-dependent primary response genes (*D*), *Nfkbiz*-dependent secondary response genes (*E*), and genes repressed by lipid A in WT BMDMs that display statistical *Nfkb1*/*Nfkbiz* codependence (*F*). Transcript levels are shown for *Nfkb1*^−/−^ and *Nfkbiz*^−/−^ BMDMs, along with WT BMDMs analyzed in parallel. Transcript levels represent averages of two biological replicates.

One possible mechanism that might contribute to p50-dependent transcription is through its reported interactions with nuclear IκB proteins (Hayden and Ghosh 2012; Chiba et al. 2013; Annemann et al. 2016). To explore a possible role for the nuclear IκBs, we performed nascent transcript RNA-seq with BMDMs from *Nfkbiz*^−/−^ (IκBζ), *Bcl3*^−/−^, and *Nfkbid*^−/−^ (IκBNS) mice. In all cases, WT and mutant cells were compared in parallel after lipid A stimulation for 0, 0.5, 1, 2, and 6 h, with two time courses performed for each WT and mutant pair. Significant differences were not observed for any genes with either *Bcl3*^−/−^ or *Nfkbid*^−/−^ BMDMs (Fig. 2A; data not shown). However, in *Nfkbiz*^−/−^ BMDMs, 136 genes exhibit strongly diminished transcript levels (WT RPKM > 3; <33% relative to WT; *P*-value < 0.01), with 30, 63, and 105 of these genes diminished at the 1, 2, and 6 h time points, respectively (Fig. 2A,B). A subset of these genes was previously shown to be IκBζ-dependent (Yamamoto et al. 2004; Chiba et al. 2013; Annemann et al. 2016).

Interestingly, 28 of the 67 genes (42%) exhibiting p50 dependence are dependent on IκBζ (Fig. 2B, red dots; Supplemental Fig. S2). In fact, p50/IκBζ codependence is observed at seven of the 11 genes (64%) exhibiting the strongest p50 dependence (*Nfkb1*^−/−^ vs. WT RPKM ratio <10%) (see vertical dashed line in Fig. 2B; Supplemental Fig. S2). Most notably, 12 of the 20 p50-dependent secondary response genes (60%) exhibit IκBζ codependence (Figs. 1I, 2C, black dots; Supplemental Fig. S2). IκBζ did not contribute to the initial induction of any of the p50-dependent primary response genes (Fig. 1D,H; Supplemental Fig. S2), consistent with the fact that the *Nfkbiz* gene is itself a primary response gene. However, all three primary response genes that exhibited delayed p50 dependence (*Il1b*, *Ccl7*, and *Slc7a2*) exhibited IκBζ codependence (Figs. 1F,J, 2C; Supplemental Fig. S2). Notably, the delayed dependence of these three genes on both p50 and IκBζ (in contrast to the early p50 dependence—in the absence of IκBζ dependence—of the other p50-dependent primary response genes) (see Supplemental Fig. S2) adds support to the notion that p50 and IκBζ are acting together to regulate this small group of primary response genes.

In addition to the genes that show clear codependence on both p50 and IκBζ for their activation, several other primary response, secondary response, weakly induced, and repressed genes exhibit IκBζ dependence without p50 codependence (Fig. 2B, blue dots; Supplemental Fig. S3). All of the primary response genes that exhibit strong dependence only on IκBζ exhibit IκBζ dependence almost exclusively at late times after their initial primary response activation (Fig. 2D; Supplemental Fig. S3). As shown in Figure 2D, transcript levels for some genes that are dependent only on IκBζ are also reduced in *Nfkb1*^−/−^ cells but by a smaller magnitude that does not meet our dependence criteria. Secondary response genes dependent only on IκBζ included two prominent cytokine genes, *Il12b* and *Il10*, and the serum amyloid gene *Saa3* (see Fig. 2E for examples; Supplemental Fig. S3). Finally, the weakly induced and repressed genes that exhibit IκBζ dependence generally exhibit dependence for their sustained transcription at late time points (see Fig. 2F for examples; Supplemental Fig. S3). Notably, several genes exhibiting p50 and/or IκBζ dependence exhibit both primary and secondary response characteristics (i.e., early CHX-independent induction followed by later CHX-dependent superinduction) (data not shown).

To summarize, p50/IκBζ codependence is observed at 42% of p50-dependent genes, including 64% of the genes exhibiting the strongest p50 dependence. The high prevalence of codependence is surprising given the diverse mechanisms by which p50 is thought to contribute to gene transcription. An interest in better understanding the close collaboration between p50 and IκBζ is heightened by the frequent relationship between *NFKBIZ* and human diseases, including cancer and ulcerative colitis (e.g., Kakiuchi et al. 2020; Nanki et al. 2020).

### Analysis of *Nfkb2*^−/−^ and *Nfkb1*^−/−^*Nfkb2*^−/−^ macrophages

Many genes activated by p50-containing dimers may not exhibit p50-dependent transcription due to redundancy with p50's close paralog, p52. Redundancy with p52 may also contribute to the existence of a set of IκBζ-dependent/p50-independent genes. To gain insight into the contribution of p52, we first performed RNA-seq with *Nfkb2*^−/−^ BMDMs. The results revealed a small number of p52-dependent genes. However, none of these genes exhibited IκBζ dependence (data not shown).

We were unable to successfully breed *Nfkb1*^−/−^*Nfkb2*^−/−^ mice, consistent with prior evidence of poor viability (Franzoso et al. 1997a). Therefore, to examine redundancy between p50 and p52, we created a J2 retrovirus immortalized macrophage line from *Nfkb1*^−/−^ mice and then used CRISPR–Cas9 editing to disrupt the *Nfkb2* gene in this line (Supplemental Fig. S4). mRNA-seq analysis comparing the *Nfkb1*^−/−^*Nfkb2*^−/−^ line and the parental *Nfkb1*^−/−^ line with a WT J2 transformed macrophage line revealed that the combined p50 and p52 deficiency yields reduced transcript levels at a larger number of inducible genes than observed in the absence of p50 alone (Supplemental Fig. S4D). However, ∼70% of strongly induced genes remain largely unaffected (Supplemental Fig. S4), either because they can be activated by NF-κB dimers that lack p50 and p52 or because their induction is entirely NF-κB-independent. Notably, only a few additional IκBζ-dependent/p50-independent genes exhibit reduced transcript levels in the *Nfkb1*^−/−^*Nfkb2*^−/−^ line (Supplemental Fig. S4D). This result demonstrates that the p50 independence of IκBζ-dependent/p50-independent genes is not generally due to redundancy between p50 and p52.

### Initial p50, IκBζ, and RelA ChIP-seq analyses

**Figure 3.**
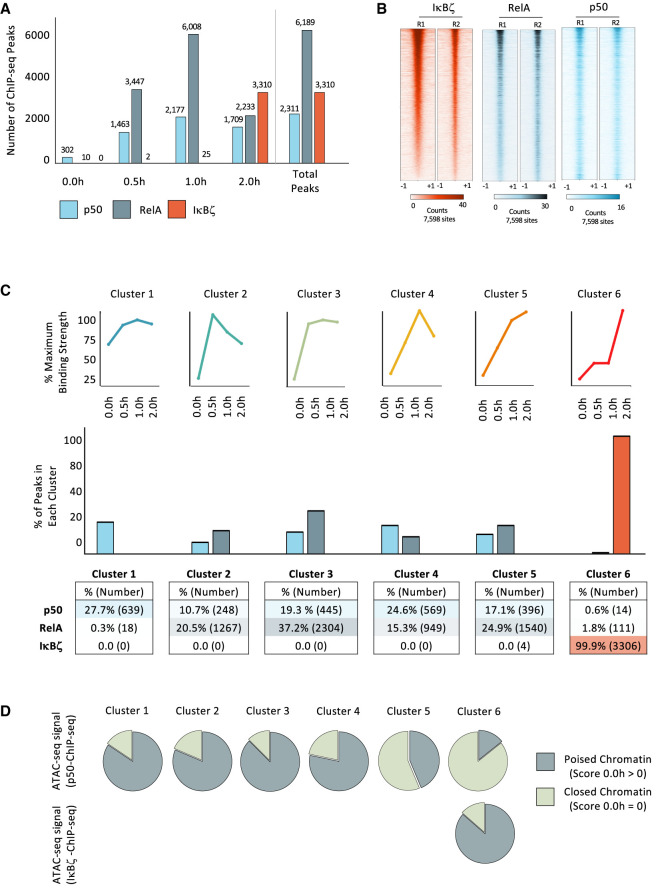
RelA, p50, and IκBζ ChIP-seq analysis. (*A*) The number of reproducible binding sites (PS > 19 and RPKM > 3 in two out of two replicates) at each time point (0, 0.5, 1, and 2 h) is plotted. The total number of unique sites for each protein is shown at the *right*. (*B*) Heat maps are shown from two biological replicates of ChIP-seq experiments for IκBζ, RelA, and p50 in cells stimulated for 2 h. The heat maps contain 7598 peaks, which include all unique peaks bound by RelA, p50, or IκBζ. All heat maps are ranked in descending order based on the binding strength in IκBζ replicate 1 (R1). (*C*) IκBζ, RelA, and p50 ChIP-seq peaks (average RPKM of two replicates for each protein at each time point) were combined and examined by *k*-means cluster analysis to identify six kinetic clusters. (*Top*) Line graphs are shown to represent the six clusters. (*Middle*) The percentages of ChIP-seq peaks for p50, RelA, or IκBζ in each of the six kinetic clusters are shown in bar graphs. (*Bottom*) Percentages and numbers are shown. The bar graph is color-coded as in *A*. (*D*) Pie graphs at the *top* show the fraction of p50 ChIP-seq peaks in each of the six kinetic clusters found at regions that exhibit either chromatin accessibility or inaccessibility by ATAC-seq (i.e., called ATAC-seq peaks; peak score of >0 or 0, respectively) in unstimulated BMDMs. The pie graph at the *bottom* shows that, although IκBζ binding always aligns with kinetic cluster 6, locations bound by IκBζ are usually accessible in unstimulated cells (i.e., poised chromatin).

To help elucidate the mechanisms underlying the regulation of p50/IκBζ-codependent genes, we performed ChIP-seq with p50, RelA, and IκBζ antibodies in BMDMs stimulated with lipid A for 0, 0.5, 1, and 2 h. We observed 2311, 6189, and 3310 reproducible peaks (peak score [PS] > 19 and RPKM > 3 in two out of two biological replicates at one or more time points) with p50, RelA, and IκBζ antibodies, respectively (Fig. 3A, right). The different peak numbers may be due to different antibody qualities and/or different numbers of genomic interactions. Despite the different peak numbers, extensive overlap was observed (Fig. 3B). Importantly, the large number of IκBζ and p50 peaks demonstrates that p50/IκBζ-codependent and IκBζ-dependent transcription is not simply due to highly selective genomic interactions.

To better understand the relationships between the three proteins, we compared peak numbers at each time point (Fig. 3A, left). In unstimulated cells, 302 p50 peaks were observed, consistent with evidence that only p50 homodimers localize to the nucleus prior to stimulation (Kang et al. 1992; Zhong et al. 2002). At 0.5 and 1 h after stimulation, increasing numbers of p50 and RelA peaks were observed. In contrast, IκBζ peaks are largely restricted to the 2 h time point, consistent with the time needed to induce IκBζ expression (Fig. 3A).

### IκBζ binds genomic sites bound earlier by NF-κB

Next, by *k*-means cluster analysis, we classified all p50, RelA, and IκBζ binding sites into six distinct kinetic clusters (Fig. 3C, top). Cluster 1, representative of peaks first observed in unstimulated cells, is apparent only at a subset (639; 27.7%) of the p50 peak profiles (Fig. 3C, middle and bottom). Substantial percentages of both the p50 and RelA peak profiles are highly prevalent in clusters 2–5, where strong binding is first observed at either 0.5 or 1 h (Fig. 3C). In contrast, IκBζ peaks are restricted almost entirely (99.9%) to cluster 6, where binding is low prior to 2 h (Fig. 3C). Notably, <2% of p50 and RelA peaks aligned with this kinetic profile (Fig. 3C). Thus, given the extensive overlap between peaks, the results suggest that IκBζ typically binds genomic sites bound at earlier time points by NF-κB dimers (see below).

Next, we used ATAC-seq to examine the chromatin state of sites bound by p50, RelA, and IκBζ. Most p50 peaks within clusters 1–4 are found at sites with ATAC-seq peaks in unstimulated cells (Fig. 3D), consistent with extensive evidence that NF-κB dimers often bind regions assembled into poised chromatin (Ghisletti et al. 2010; Heinz et al. 2010). In contrast, p50 peaks within clusters 5 and 6, which exhibit relatively late p50 binding, are found at sites that lack ATAC-seq peaks in unstimulated cells (Fig. 3D). This finding suggests that the delayed p50 binding in these clusters is associated with a frequent need for nucleosome remodeling prior to NF-κB binding. Importantly, although IκBζ peaks align almost exclusively with cluster 6, they are found at sites that exhibit ATAC-seq accessibility in unstimulated cells (Fig. 3D, bottom, cluster 6). This finding further strengthens the notion that IκBζ typically binds regions already assembled into open chromatin and already associated with an NF-κB dimer. These findings are consistent with a prior study performed with representative genes (Kayama et al. 2008).

### Analysis of preferential genomic interactions by p50 and RelA

**Figure 4.**
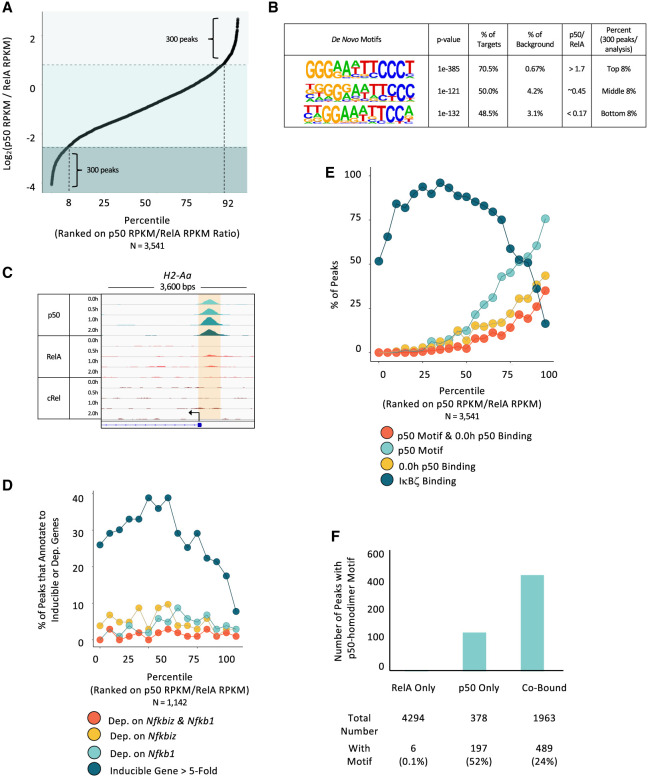
Relationship between preferential p50 or RelA binding and transcriptional dependence. (*A*) All reproducible p50 binding sites (PS > 19 and RPKM > 3 in two out of three replicates; 2311 peaks) and the top 2311 reproducible RelA binding sites were combined (total of 3541 peaks) and ordered based on the 1 h average p50 RPKM:RelA RPKM ratio. The 300 peaks (8% of the total) with the strongest preference for either p50 (*top right*) or RelA (*bottom left*) are indicated. (*B*) De novo motif analyses were performed with sequences underlying the 300 peaks with the strongest p50 preference (top 8%), the strongest RelA preference (bottom 8%), and no preference (middle 8%). (*C*) Browser tracks at the *H2-Aa* locus show an example of p50 preferential binding. (*D*) Each ChIP-seq peak from *A* was annotated to its closest gene. Peaks located within 5 kb of the TSS for a gene (1142 peaks) were included in this analysis, and the remaining peaks were excluded. The included peaks were divided into 15 equal bins. The percentages of peaks in each bin that annotate to a gene induced by lipid A by more than fivefold are in dark blue, revealing that peaks preferentially bound by p50 infrequently annotate to inducible genes. The percentages of peaks in each bin that annotate to *Nfkb1/Nfkbiz*-codependent (red), *Nfkbiz*-dependent (gold), and *Nfkb1*-dependent (light blue) genes are also shown, revealing no correlation with preferential binding. (*E*) p50 and RelA ChIP-seq peaks from *A* were divided into 20 equal bins. The percentages of peaks in each bin that coincide with an IκBζ ChIP-seq peak are in dark blue, demonstrating that IκBζ binds infrequently to regions displaying p50 preferential binding. The percentage of peaks in each bin that exhibit p50 ChIP-seq peaks in unstimulated cells is shown in gold. The percentage of peaks in each bin that contain the motif that most strongly supports p50 homodimer binding (three G:C base pairs in each half-site separated by 5 bp) is shown in light blue, and the percentage of peaks that both contain a p50 homodimer motif and display p50 binding in unstimulated cells is in red. (*F*) The numbers of genomic locations that exhibit reproducible peaks (PS > 19 and RPKM > 3 in two out of three samples) for only RelA (4294), only p50 (378), and both p50 and RelA (1963) are shown, along with the number of peaks in each group that contain a p50 homodimer motif (three G:C base pairs in each half-site separated by 5 bp). This analysis shows that, although the probability of finding a p50 homodimer motif at a location that displays only a p50 ChIP-seq peak is the highest (52% of these peaks), a larger number of locations with both p50 and RelA ChIP-seq peaks contain p50 homodimer motifs (489) than locations with only a p50 ChIP-seq peak (197).

To further elucidate the characteristics of sites bound by these proteins, we asked whether IκBζ binding correlates with preferential binding by p50 or RelA. We first selected the strongest reproducible 2311 peaks from the p50 and RelA ChIP-seq experiments, which generated 3541 total peaks when combined. We then calculated the p50/RelA RPKM ratio at each region containing a called peak for either protein (Fig. 4A).

Motif enrichment analysis was performed with the 300 peaks representing either the largest or smallest p50:RelA RPKM ratios, as well as the 300 peaks with intermediate ratios (Fig. 4B). The bin with the greatest p50 preferential binding (Fig. 4B, top) is enriched in motifs representative of p50 homodimer binding, with three G:C base pairs in each half-site (structural studies revealed that p50 subunits strongly associate with three G:C base pairs within a half-site, whereas RelA associates with only two G:C base pairs) (Huxford and Ghosh 2009). The bin with the greatest RelA preferential binding (Fig. 4B, bottom) is enriched in motifs representative of RelA homodimer binding, with two G:C base pairs in each half-site. The bin with intermediate RPKM ratios (Fig. 4B, middle) is enriched in motifs that less clearly represent either p50 or RelA interactions. Figure 4C shows browser tracks displaying an example of a p50 preferential site at the *H2-Aa* promoter.

We next divided the ChIP-seq peaks from Figure 4A into 15 equal bins and calculated the percentage of peaks within each bin that annotate to a TLR4-induced gene (fold induction >5) using a nearest gene approach (Fig. 4D, dark blue). Interestingly, ChIP-seq peaks that exhibit the greatest p50 preferential binding (Fig. 4D, right, dark blue) are the least likely to annotate to induced genes, suggesting that p50 homodimer interactions do not typically contribute to TLR4-induced transcription.

p50 preferential binding also correlates with an increased probability of p50 binding in unstimulated cells. Specifically, in the bin displaying the greatest p50 preferential binding, 43% of peaks are at locations exhibiting p50 peaks in unstimulated cells (Fig. 4E, gold). Furthermore, in this bin, 76% of the peaks coincide with a motif expected to support p50 homodimer binding (Fig. 4E, light blue), with successively smaller percentages in bins with lower p50/RelA binding ratios. Overall, 35% of peaks in the bin with the strongest p50 preferential binding both contain a homodimer motif and exhibit a p50 peak in unstimulated cells (Fig. 4E, red).

The above results reveal that p50 homodimer motifs are prevalent at sites displaying p50 preferential binding in stimulated cells. However, although the probability of finding a p50 homodimer motif is highest in bins displaying p50 preferential binding, this represents a relatively small number of p50 binding sites with p50 homodimer motifs. Specifically, 197 of 378 (52%) of the p50 called peaks that lack RelA called peaks contain a p50 homodimer motif (Fig. 4F). In comparison, 489 of 1963 (24%) of the p50 called peaks that also display a RelA called peak contain a p50 homodimer motif. Thus, a p50 homodimer motif does not strongly predict p50 preferential binding. One possibility is that, following cell stimulation, the abundance of RelA:p50 heterodimers greatly exceeds the abundance of p50 homodimers, leading to RelA:p50 heterodimers binding to homodimer motifs.

To summarize, the results in Figure 4 demonstrate that p50 preferential and RelA preferential binding can be observed at motifs predicted to represent binding of p50 homodimers or RelA homodimers, respectively. p50 preferential peaks are least likely to be near a lipid A-induced gene but are the most likely to correlate with p50 binding in unstimulated cells. Moreover, the presence of a p50 homodimer motif does not predict the presence of p50 preferential binding.

### IκBζ genomic interactions and IκBζ-dependent genes are enriched at regions that support p50 and RelA cobinding

We next explored the prevalence of IκBζ ChIP-seq peaks across the RelA–p50 binding ratio profile. This analysis reveals that IκBζ binding is enriched at sites that support binding by both p50 and RelA (p50:RelA ratio of 0.3–1.6) and is observed much less frequently in bins with either a strong p50 or a strong RelA preference (Fig. 4E, dark blue). This result suggests that IκBζ may not preferentially bind p50 homodimers.

To examine the relationship between preferential binding and transcription, we returned to the gene dependence classifications from Figure 2B and asked whether p50/IκBζ-codependent, IκBζ-dependent, and p50-dependent genes correlate with genomic regions that exhibit preferential p50 binding. For this analysis, each peak was annotated to its closest gene (but only if a gene's TSS is located within 5 kb of the ChIP-seq peak, to increase confidence in the assignments), and the prevalence of peaks annotating to p50/IκBζ-, IκBζ-, and p50-dependent genes was analyzed across the spectrum of p50–RelA RPKM ratios. This analysis revealed that peaks near all three gene classes are broadly distributed, with no preference toward peaks preferentially bound by either RelA or p50 (Fig. 4D). These results add further support to the notion that p50 homodimers do not play a central role in selective gene regulation by IκBζ or in the regulation of p50-dependent genes.

### p50- and IκBζ-dependent genes correlate with p50-dependent IκBζ genomic interactions

**Figure 5.**
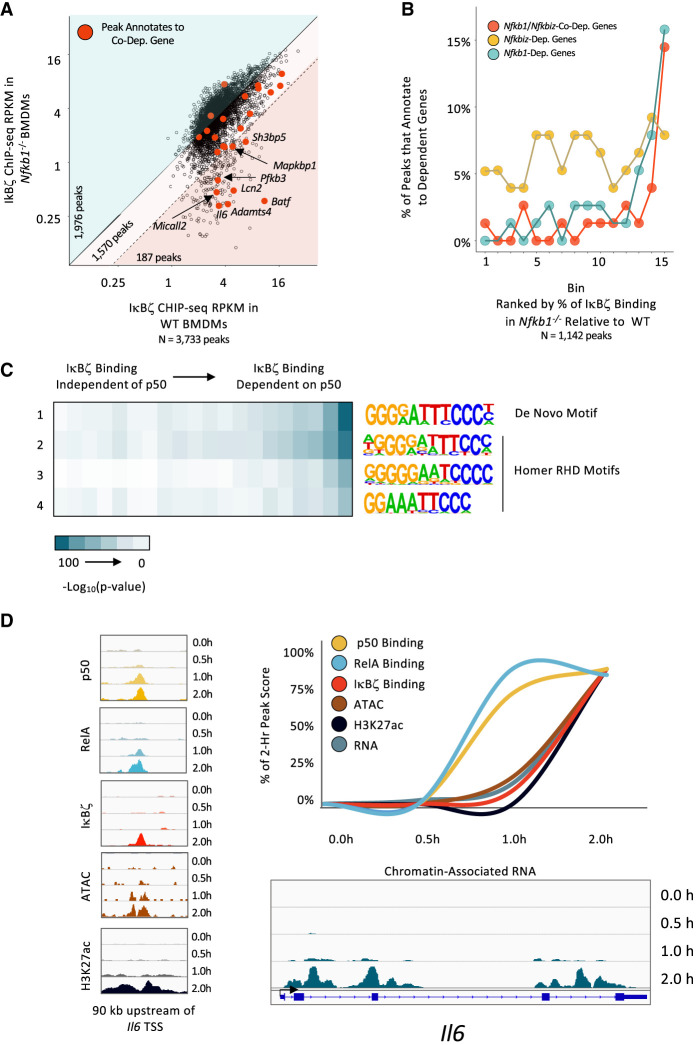
p50 dependence of IκBζ binding and time course of events. (*A*) For all IκBζ ChIP-seq peaks (PS > 19 and RPKM > 3 in two out of three replicates) in WT macrophages stimulated with lipid A for 2.0 h, the average RPKM in WT (*x*-axis) versus *Nfkb1*^−/−^ (*y*-axis) macrophages is plotted. The blue background highlights 1976 peaks with >100% IκBζ RPKM in *Nfkb1*^−/−^ versus WT macrophages. The light-pink background highlights 1570 IκBζ binding sites with a mild dependence on *Nfkb1* (33%–100% relative to WT). The pink background highlights 187 IκBζ peaks (5% of total) with a strong dependence on p50 (<33% binding strength relative to WT). Red dots indicate binding sites that annotate to *Nfkb1*/*Nfkbiz*-codependent genes. Although multiple peaks may annotate to a single gene, only one peak per gene is highlighted in red: the peak with the maximum change in IκBζ binding between *Nfkb1*^−/−^ and WT BMDMs. (*B*) Quantification of the results in *A* is shown. IκBζ peaks (PS > 19 and RPKM > 3 in two out of three replicates; restricted to peaks within 30 kb of a gene expressed at >3 RPKM, with only one peak included per gene based on greatest *Nfkb1* dependence of IκBζ binding) were placed in 15 bins based on relative dependence on *Nfkb1*. In each bin, the percentages of peaks that annotate to an *Nfkb1*/*Nfkbiz*-codependent (red), *Nfkbiz-*dependent (gold), and *Nfkb1*-dependent (blue) gene are plotted. (*C*) Motif analysis is shown for IκBζ peaks placed into 20 bins based on relative dependence on p50. Three NF-κB motifs from HOMER and one de novo motif were used. The heat map corresponds to the −log_10_(*P*-value) for motif enrichment. (*D*) Properties of a representative p50-dependent IκBζ binding site located 90 kb upstream of the *Nfkb1*/*Nfkbiz*-codependent *Il6* gene are displayed. Genome browser tracks at the *left* show ChIP-seq tracks for p50, RelA, IκBζ, and H3K27ac and ATAC-seq tracks at 0, 0.5, 1, and 2 h after lipid A stimulation. The ChIP-seq, ATAC-seq, and chromatin-associated transcript RNA-seq data are quantified in the line graph at the *top right* using peak scores for ChIP-seq and ATAC-seq data and RPKM for RNA-seq data. Genome browser tracks showing chromatin-associated transcript data for the *Il6* gene are shown at the *bottom right*.

The above analyses provided important insights but failed to reveal the mechanisms underlying dependence on p50 and/or IκBζ. We therefore extended our analysis by examining the p50 dependence of IκBζ genomic interactions. For this analysis, IκBζ ChIP-seq was performed in parallel with WT and *Nfkb1*^−/−^ BMDMs. Surprisingly, only ∼5% of IκBζ genomic interactions (187 peaks) exhibited strong dependence (<33% binding of IκBζ in *Nfkb1*^−/−^ cells relative to WT cells) on p50 (Fig. 5A).

To evaluate the possible significance of this small set of p50-dependent IκBζ binding events, we examined their locations in relation to p50/IκBζ-, IκBζ-, and p50-dependent genes. Interestingly, IκBζ ChIP-seq peaks near p50–IκBζ-codependent and p50-dependent genes are frequently among the small fraction of p50-dependent IκBζ ChIP-seq peaks (Fig. 5A, peaks near codependent genes in red). To quantify this finding, IκBζ ChIP-seq peaks located within 30 kb of an expressed gene were separated into 15 bins on the basis of their magnitude of dependence. The percentages of peaks within each bin that annotate to genes with transcriptional p50/IκBζ codependence, p50 dependence, and IκBζ dependence were then calculated. In the bin containing IκBζ peaks that exhibit the strongest requirement for p50 (bin 15), 14% of the peaks annotate to genes with transcriptional p50/IκBζ codependence, 16% annotate to genes with transcriptional p50 dependence, and 8% annotate to genes with transcriptional IκBζ dependence (Fig. 5B). Thus, in total, a remarkable 38% of peaks in the bin where IκBζ exhibits the strongest binding dependence on p50 annotate to a gene exhibiting transcriptional dependence on p50 and/or IκBζ. For transcriptional p50/IκBζ codependence and p50 dependence, this represents a large enrichment, as <3% of IκBζ peaks in bins 1–12 annotate to either p50/IκBζ-codependent or p50-dependent genes. These results demonstrate that although IκBζ genomic interactions can be observed by ChIP-seq at thousands of genomic locations, we can enrich for those that support p50/IκBζ-codependent or p50-dependent transcription by examining the dependence of IκBζ binding on p50.

The above results suggest that p50 dependence of IκBζ binding may be predictive of a specific interaction or a binding conformation that supports IκBζ function (see the Discussion). To explore these possibilities, we compared binding strengths, p50 versus RelA binding preferences, and binding motifs between p50-dependent and p50-independent IκBζ genomic interaction sites. No differences in binding strengths or binding preferences were observed (data not shown).

To compare motif enrichment at p50-dependent versus p50-independent IκBζ binding sites, we separated all IκBζ binding sites into 20 bins based on their magnitude of p50 dependence. We then examined the enrichment of four NF-κB motifs in each bin (three NF-κB motifs from the HOMER motif software and a de novo motif generated from our data). We found the strongest enrichment of all four motifs in bins containing IκBζ peaks with the greatest p50 dependence (Fig. 5C). This result suggests that functional interactions between IκBζ and NF-κB occur at NF-κB consensus motifs. The relatively low enrichment of NF-κB motifs at p50-independent IκBζ peaks raises the possibility that IκBζ binding to these sites often does not require an interaction with an NF-κB dimer. However, NF-κB co-occupies a high percentage of these sites with IκBζ (data not shown), suggesting instead that both IκBζ and an NF-κB dimer occupy these sites, often in the absence of an NF-κB consensus motif.

Finally, we explored the relationship between the dependence of IκBζ binding on p50 and H3K37ac, a histone modification frequently used as a marker of a transcriptionally active state. At a genome-wide scale, the kinetics of IκBζ binding do not correlate with the kinetics of histone H3K27ac. However, if we instead focus on the small number of IκBζ peaks that exhibit p50-dependent binding, the kinetics of IκBζ binding correlate much more closely with the kinetics of the H3K27ac modification. A putative *Il6* enhancer that displays strong p50-dependent IκBζ binding provides an example (Fig. 5D). At this enhancer, p50 and RelA peaks are readily observed at the 1 h time point, but IκBζ binding, H3K27ac, and nascent transcripts are barely detectable until 2 h. The ATAC-seq peak is not called until the 2 h time point, but an examination of the ATAC-seq tracks (Fig. 1D, left) shows that accessibility is substantially elevated by 1 h, suggesting that initial chromatin opening coincides with RelA and p50 binding. Together, these results suggest that at IκBζ binding sites that are likely to be functionally important, NF-κB-dimer binding and chromatin opening precede IκBζ binding, H3K27ac, and transcription. Thus, a kinetic relationship between IκBζ binding and the H3K27ac modification may be another feature of functionally important IκBζ binding events, along with the p50 dependence of IκBζ binding.

### In vivo and in vitro analyses of IκBζ interactions with p50 and RelA

The above findings were somewhat surprising, as prior studies proposed that IκBζ functions by contributing a transactivation domain to p50 homodimers (Yamamoto et al. 2004; Trinh et al. 2008). However, other studies demonstrated that IκBζ interacts with both RelA and p50 in cell extracts, suggestive of an interaction with RelA:p50 heterodimers (Yamazaki et al. 2001, 2008; Totzke et al. 2006; Kannan et al. 2011). Quantitative coimmunoprecipitations that we performed were consistent with the latter studies in showing comparable IκBζ interactions with both p50 and RelA (data not shown).

We also performed electrophoretic mobility shift assays (EMSAs) with extracts containing overexpressed RelA, p50, and IκBζ, either expressed individually and combined or coexpressed. As observed previously (Yamazaki et al. 2001; Totzke et al. 2006), IκBζ strongly inhibited DNA binding by both p50 homodimers and RelA:p50 heterodimers (data not shown). The inhibition of DNA binding provides further evidence that IκBζ can bind both RelA:p50 heterodimers and p50 homodimers in vitro. However, in vivo, IκBζ clearly co-occupies DNA with NF-κB dimers rather than inhibiting their binding (see the Discussion).

**Figure 6.**
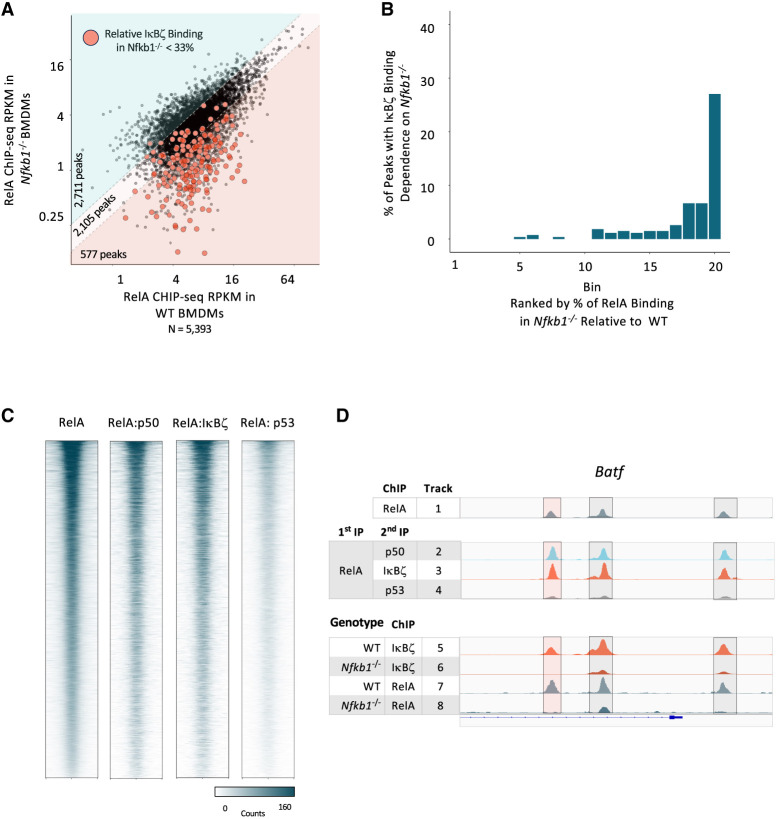
Possible relevance of RelA in IκBζ-dependent transcription. (*A*) For all RelA ChIP-seq peaks (PS > 19 and RPKM > 3 in two out of three replicates) in WT macrophages stimulated with lipid A for 2 h, the average RPKM in WT (*x*-axis) versus *Nfkb1*^−/−^ (*y*-axis) macrophages is plotted. The pink background highlights 577 RelA peaks (10.7% of total) that exhibit the smallest *Nfkb1*^−/−^ RPKM:WT RPKM ratios. Red dots indicate peaks that also display small *Nfkb1*^−/−^ RPKM:WT RPKM ratios for IκBζ binding (<0.33) (see Fig. 5A). (*B*) Quantification of the results in *A* is shown. All RelA peaks were placed in 20 bins based on relative dependence on *Nfkb1*. In each bin, the percentages of peaks that annotate to locations that also exhibit *Nfkb1*-dependent IκBζ binding are displayed, showing that *Nfkb1*-dependent RelA binding frequently coincides with *Nfkb1*-dependent IκBζ binding. (*C*) Heat maps are shown for sequential ChIP-seq peaks. The first heat map shows rank-ordered peaks obtained by RelA ChIP-seq. The other three heat maps show the same rank-ordered peaks after sequential ChIP-seq, with the first immunoprecipitation performed with RelA antibodies, and the second immunoprecipitation performed with antibodies directed against p50 (second heat map), IκBζ (third heat map), or p53 (fourth heat map, as a negative control). (*D*) Browser tracks are shown for three sites in the *Batf* locus that exhibit *Nfkb1*-dependent binding of both RelA and IκBζ. (The location highlighted in pink shows the greatest *Nfkb1* dependence.) Track 1 shows RelA ChIP-seq peaks obtained in parallel with the sequential ChIP-seq results. Tracks 2–4 show the sequential ChIP-seq results using RelA antibodies for the first precipitation and p50 (track 2), IκBζ (track 3), and p53 (track 4) antibodies for the second precipitation. Tracks 5–8 show IκBζ or RelA ChIP-seq peaks from WT and *Nfkb1*^−/−^ BMDMs, as indicated.

To further explore this issue in vivo, we performed RelA ChIP-seq in *Nfkb1*^−/−^ BMDMs. The results revealed that only a small subset of RelA binding events across the genome is dependent on p50 (Fig. 6A). Many of these binding events correspond to sites where IκBζ binding was found to be p50-dependent (Fig. 6A, pink dots). To quantify this observation, RelA ChIP-seq peaks were divided into 20 bins on the basis of their p50 dependence. The percentage of peaks in each bin that exhibit p50-dependent binding of IκBζ was then calculated (Fig. 6B). Remarkably, in the bin with RelA peaks showing the greatest p50 dependence, 27% of the peaks also exhibited p50-dependent IκBζ binding (Fig. 6B). This finding further highlights the existence of a select subset of NF-κB genomic binding sites at which both RelA and IκBζ bind in a manner that is strongly dependent on the presence of p50. This is in contrast to the vast majority of genomic sites where RelA and IκBζ binding are p50-independent.

Finally, to further examine which NF-κB dimers can support IκBζ’s association with DNA in vivo, we used sequential ChIP-seq experiments. For these experiments, we first performed ChIP with RelA antibodies. We then used 10 mM DTT to dissociate the immunoprecipitated chromatin from the antibody–bead complex, followed by a second immunoprecipitation with antibodies directed against either p50, IκBζ, or p53 (as a negative control). The results reveal that the use of p50 or IκBζ antibodies for the second immunoprecipitation resulted in comparably high efficiencies of immunoprecipitation of chromatin fragments previously immunoprecipitated with RelA antibodies, whereas p53 antibodies were much less efficient (Fig. 6C). Browser tracks for three representative peaks at the *Batf* locus show the efficiency of secondary immunoprecipitation by the p50 and IκBζ antibodies in comparison with p53 antibodies (Fig. 6D, tracks 2–4.). Notably, all three of these peaks exhibit substantial p50-dependent binding of both IκBζ and RelA (Fig. 6D, tracks 5–8). These results therefore support the hypothesis that IκBζ has the potential to bind DNA in association with RelA:p50 heterodimers.

### IκBζ is important for the differential responses to TLR4 and TNFR signaling

As described above, a small set of key inflammatory and immunoregulatory genes exhibits strong p50/IκBζ transcriptional codependence. Other key genes, including *Il12b*, exhibit strong dependence on one or both factors in lipid A-stimulated macrophages. To explore the potential biological significance of the p50/IκBζ regulatory pathway, we searched for settings in which IκBζ might contribute to differential transcription. This effort led to a focus on BMDMs stimulated with lipid A versus TNF. Although *Nfkbiz* is a primary response gene that is potently activated by lipid A, previous studies have shown that the *Nfkbiz* gene is poorly expressed in TNF-stimulated macrophages (Yamazaki et al. 2001, 2005).

**Figure 7.**
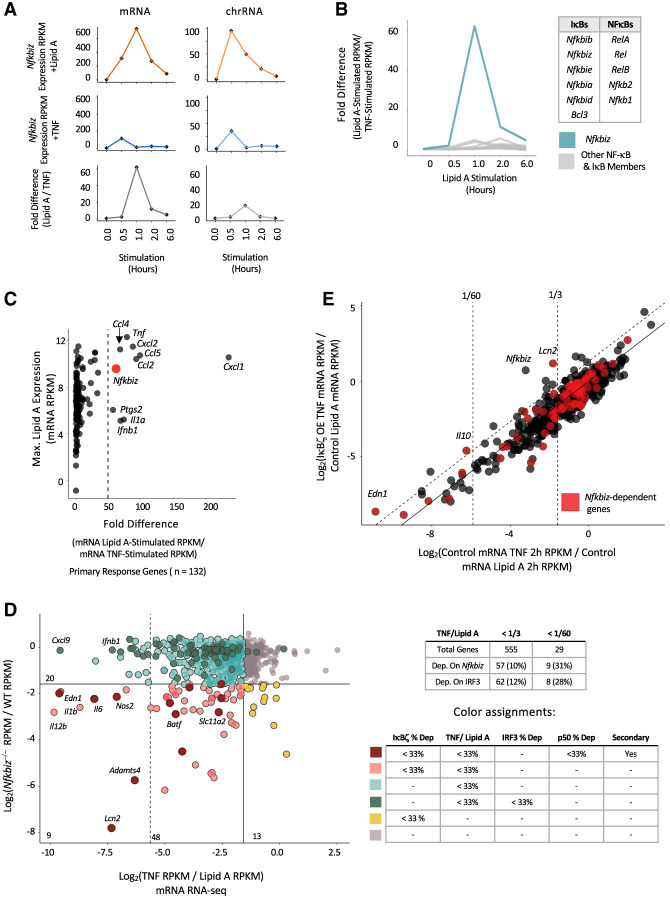
Contribution of IκBζ to differential BMDM responses to lipid A and TNFα. (*A*) The line graphs show *Nfkbiz* transcript levels (RPKM) in stimulated BMDMs at each of five stimulation time points (0, 0.5, 1, 2, and 6 h) from mRNA-seq (*left*) and nascent chromatin-associated transcript RNA-seq (*right*). Values are shown for lipid A (*top*) and TNF (*middle*) stimulation, with the lipid A:TNF ratio (fold difference) at each time point at the *bottom*. (*B*) The fold differences (ratios) between lipid A- and TNF-stimulated RPKM are shown at each time point for all five NF-κB and six IκB family members. *Nfkbiz* is highlighted in blue. (*C*) All 132 strongly induced (FC > 10) primary response genes (Tong et al. 2016) are plotted based on maximum mRNA transcript levels (RPKM) in lipid A-stimulated macrophages (*y*-axis) versus the fold difference (ratio) in mRNA transcript levels between lipid A- and TNF-stimulated BMDMs (*x*-axis). The dashed line corresponds to a 50-fold difference*. Nfkbiz* is among only nine primary response genes that differ >50-fold. (*D*) A scatter plot showing that a large fraction of genes exhibiting the greatest differential transcription between lipid A- and TNF-stimulated BMDMs also exhibit strong *Nfkbiz*-dependent transcription. All 802 expressed and inducible genes (lipid A induction >3; WT RPKM > 3) are plotted. The *y*-axis shows the ratio (log_2_) of chromatin-associated transcript levels (RPKM) in lipid A-stimulated *Nfkbiz*^−/−^ BMDMs versus lipid A-stimulated WT BMDMs. The *x*-axis shows the ratio of mRNA levels (RPKM) from TNF-stimulated versus lipid A-stimulated BMDMs. Vertical solid and dashed lines represent TNF versus lipid A differential transcript ratios of 1:3 (*right*) and 1:60 (*left*), respectively. The horizontal solid line represents an *Nfkbiz*^−/−^:WT ratio of 1:3. Genes that exhibit both *Nfkbiz* dependence and differential TNF versus lipid A transcript levels are in pink. Genes that exhibit *Nfkbiz* and *Nfkb1* dependence, display differential TNF versus lipid A transcript levels, and are secondary response genes are in dark red. Genes that are only *Nfkbiz*-dependent or that only display differential TNF versus lipid A transcript levels are in gold and light blue, respectively (see detailed color assignments at the *bottom right*). Note that nine out of 29 genes (31%) that exhibit the greatest differential TNF versus lipid A transcript levels (to the *left* of the *left*-most vertical dashed line) exhibit *Nfkbiz* dependence. The table at the *right* shows the numbers of genes that exhibit *Nfkbiz* dependence and IRF3 dependence among those genes that exhibit weak (<1:3) or strong (<1:60) TNF versus lipid A differential expression. (*E*) A scatter plot showing the impact of IκBζ overexpression on gene transcription in TNF-stimulated macrophages (a J2 transformed macrophage line). On the *y*-axis, for all lipid A-inducible genes (induction greater than threefold; WT RPKM > 3), ratios of mRNA levels (RPKM) in TNF-stimulated macrophages transduced with an *Nfkbiz*-expressing retrovirus versus lipid A-stimulated untransduced macrophages are plotted (2 h stimulation time point). On the *x*-axis, ratios of mRNA levels in TNF-stimulated macrophages versus lipid A-stimulated macrophages (2 h time points) are plotted. Vertical dashed lines correspond to TNF versus lipid A differential transcript levels of 1:60 (*left*) and 1:3 (*right*). The diagonal solid line corresponds to unchanged gene expression in the presence of IκBζ overexpression. The diagonal dashed line corresponds to 2.5-fold higher transcript levels with TNF stimulation in the presence of IκBζ overexpression compared with untransfected cells stimulated with TNF. Genes that exhibit *Nfkbiz-*dependent expression (fold change <33%; *P*-value < 0.01) are highlighted in red. The *Lcn2* gene, which is impacted to the greatest extent by IκBζ overexpression, and the *Nfkbiz* gene, which is overexpressed from the retroviral vector, are noted.

To extend the characterization of *Nfkbiz* differential expression, we performed nascent transcript RNA-seq and mRNA-seq with TNF-stimulated BMDMs. A comparative analysis reveals a large magnitude of *Nfkbiz* differential expression between TNF and lipid A stimulation. *Nfkbiz* nascent transcripts and mRNA are 18-fold and 62-fold more abundant, respectively, after lipid A stimulation than after TNF stimulation at the 1 h time point (Fig. 7A). The greater magnitude of the mRNA difference is likely due to a previously described instability of the *Nfkbiz* mRNA following TNF stimulation (Yamazaki et al. 2005).

The large magnitude of *Nfkbiz* differential expression is unusual. For example, *Nfkbiz* mRNA transcripts exhibit a differential expression magnitude that is much greater than that of all other NF-κB and IκB members (Fig. 7B). Furthermore, among all 132 strongly induced primary response genes activated by lipid A or TNF (Tong et al. 2016), *Nfkbiz* ranks ninth, with the others primarily corresponding to chemokines and cytokines rather than transcriptional regulators (Fig. 7C). These results suggest that mechanisms have emerged to ensure that the abundance of IκBζ remains extremely low following TNF stimulation.

Consistent with the strongly differential expression of *Nfkbiz*, all p50/IκBζ-codependent secondary response genes, including *Il6*, *Lcn2*, *Adamts4*, and *Nos2* (Fig. 7D, left, maroon), and 81% of the IκBζ-dependent genes, including *Il12b* (Fig. 7D, left, pink), exhibit strong differential mRNA levels (<33% TNF:lipid A RPKM ratio). The three codependent primary response genes, including *Il1b*, also exhibit strong differential transcript levels (Fig. 7D, left, pink). In fact, among the genes showing the strongest differential expression (TNF:lipid A percent expression of <2%), 31% are among the small group of p50/IκBζ or IκBζ targets (Fig. 7D, genes to the left of the dashed vertical line).

To estimate the relative contributions of IRF3 and IκBζ to the differential responses of BMDMs to lipid A versus TNF, we used nascent transcript RNA-seq data sets from *Ifnar*^−/−^ and *Irf3*^−/−^ BMDMs to determine the number of genes that exhibit strong (<33% relative to WT) dependence on IRF3 or its target, *Ifnb1*. This analysis revealed that 12% of lipid A versus TNF differentially expressed genes exhibit IRF3/IFNAR dependence (Fig. 7D, top right). In comparison, 10% of lipid A versus TNF differentially expressed genes exhibit IκBζ dependence (Fig. 7D, top right). Focusing on the 29 genes that exhibit the strongest lipid A:TNF differential expression (mRNA level in response to TNF <2% of the level in response to lipid A), we found that IRF3/IFNAR dependence can account for the differential expression of eight (28%) of these genes; in contrast, IκBζ dependence can account for the differential expression of 11 (31%) of the genes (Fig. 7D).

Finally, to determine whether an increased abundance of IκBζ is sufficient to rescue transcript levels for IκBζ-dependent genes in response to TNF stimulation, we overexpressed IκBζ in BMDMs by transduction of an IκBζ-expressing retrovirus, followed by stimulation with lipid A or TNF. In the presence of overexpressed IκBζ, the induction magnitude of one IκBζ-dependent gene, *Lcn2*, after TNF stimulation was comparable with the induction magnitude observed after lipid A stimulation (Fig. 7E). However, the induction of other p50/IκBζ-codependent and IκBζ-dependent genes was not impacted by IκBζ overexpression (Fig. 7E). One possible reason for the limited impact of IκBζ overexpression is that IκBζ activity may often require complex regulatory mechanisms, such as post-translational modifications, that cannot be achieved with the overexpressed protein. Notably, the one rescued gene, *Lcn2*, exhibits the strongest dependence on both p50 and IκBζ of all inducible genes (see Fig. 2C); this unusually strong dependence may be related to its unique ability to be rescued by IκBζ overexpression.

## Discussion

We performed a genomics-centric analysis of selective transcriptional regulation in stimulated BMDMs, with a focus on the NF-κB p50 protein and the nuclear IκB protein IκBζ. We first identified a small group of key immunoregulatory genes that exhibited a strong dependence on p50 in lipid A-stimulated BMDMs. Strong overlap between p50-dependent and IκBζ-dependent genes revealed that a defined p50/IκBζ pathway makes a major contribution to the regulation of this key set of genes. Although p50, RelA, and IκBζ occupy thousands of genomic sites, a defining characteristic of binding events that are potentially functionally meaningful was the p50 dependence of IκBζ and RelA binding. Similar temporal kinetics of IκBζ binding and H3K27ac deposition also appeared to distinguish functional from nonfunctional IκBζ interactions. In vivo and in vitro results provided strong support for the possibility that IκBζ functionally interacts with dimers containing p50 and RelA, possibly RelA:p50 heterodimers. Biologically, this pathway appears to make a major contribution to the differential responses of macrophages to lipid A and TNF.

The small number of p50-dependent genes in BMDMs and the strong overlap with IκBζ-dependent genes were unexpected, given that p50 also contributes to stimulus-induced transcription as a component of the abundant RelA:p50 and c-Rel:p50 heterodimers. Redundancy between p50 and its closely related paralog, p52, provides a partial explanation for the limited number of p50-dependent genes. However, the induction of most lipid A-induced genes was retained in *Nfkb1*^−/−^*Nfkb2*^−/−^ macrophage lines, suggesting that NF-κB dimers lacking p50 and p52 are sufficient for the induction of many genes.

Prior studies have shown that IκBζ binds p50 (Yamazaki et al. 2001; Yamamoto et al. 2004; Trinh et al. 2008; Kohda et al. 2014), but the IκBζ dependence of a high percentage of genes exhibiting p50 dependence was not previously known. A subset of prior studies of IκBζ have suggested that its function involves association with p50 homodimers (Yamamoto et al. 2004; Trinh et al. 2008), with IκBζ providing an activation domain to a homodimer that otherwise would lack such a domain. However, our sequential ChIP-seq and biochemical results are more consistent with other prior studies that revealed in vitro interactions between IκBζ and both p50 and RelA (Yamazaki et al. 2001, 2008; Totzke et al. 2006; Kannan et al. 2011).

If RelA:p50 heterodimers carry out functional interactions with IκBζ, a mechanistic understanding of IκBζ’s role would require further exploration because RelA:p50 heterodimers possess RelA's transactivation domain. According to combinatorial principles of gene regulation, the transcriptional activation of most if not all genes is thought to require multiple transcription factors, many with transactivation domains, and little is currently known about the mechanisms underlying the requirement for multiple transactivation domains for transcriptional activation. Thus, mechanistically, it is not unreasonable to envision a critical requirement for IκBζ for transcriptional activation when associated with a RelA:p50 heterodimer.

Another unanswered question is why recombinant IκBζ appears to be incapable of binding DNA-associated p50 homodimers or RelA:p50 heterodimers in EMSA experiments despite compelling evidence that it can associate with DNA-bound NF-κB dimers in vivo. This finding, observed by us and others (Yamazaki et al. 2001; Totzke et al. 2006), suggests that IκBζ may require a post-translational modification, a processing event, or the presence of another protein to allow association with DNA-bound NF-κB, raising the possibility of another layer of regulation of the p50/IκBζ pathway.

Although our data reveal strong overlap between p50-dependent and IκBζ-dependent genes, a substantial number of genes exhibit strong dependence on only one of these two proteins. IκBζ-dependent/p50-independent genes may collaborate with other NF-κB dimers or may contribute to gene regulation in concert with different transcription factor families. p50-dependent/IκBζ-independent transcription presumably reflects a requirement at a small set of genes for a p50 dimeric species that does not require IκBζ for its function.

The key immunoregulatory genes that exhibit p50/IκBζ codependence, including *Il6* and *Il1b*, are potently activated in a large number of cell types in response to diverse stimuli. p50 and IκBζ may be universally required for the activation of these genes. However, many genes are regulated by different factors in different biological settings, raising the possibility that the regulatory mechanisms uncovered here are unique to specific cell types and/or stimuli.

Finally, the finding that the *Nfkbiz* gene and most IκBζ target genes stand out due to their very large magnitude of differential expression between lipid A- and TNF-stimulated cells highlights the potential biological importance of the differential expression of these genes. The unusually low expression of *Nfkbiz* in TNF-stimulated macrophages appears to be due to the combined influence of a transcriptional mechanism that is unique among primary response genes and an mRNA stability mechanism that also appears unusual or unique (Yamazaki et al. 2005). The well-documented importance of many IκBζ target genes for antimicrobial responses provides justification for their potent activation by lipid A, but the reason transcriptional and post-transcriptional mechanisms evolved to ensure that these genes remain largely silent in response to TNF signaling is less obvious. Nevertheless, the large percentage of IκBζ-dependent genes that exhibit differential expression in response to lipid A versus TNF, and the unusually large magnitudes of their differential expression in comparison with the magnitudes observed with IRF3-dependent genes, suggest that IκBζ and the p50/IκBζ pathway are major contributors to differential lipid A/TNF gene induction.

## Materials and methods

### Mice

The *Nfkb1*^−/−^ and *Nfkb2*^−/−^ mice were a gift from Alexander Hoffmann (University of California, Los Angeles). The *Nfkbiz*^−/−^ (Yamamoto et al. 2004), *Bcl3*^−/−^ (Franzoso et al. 1997b), and *Nfkbid*^−/−^ (Touma et al. 2007) mice, all bred onto a C57BL/6 background, were kindly provided by Giorgio Trinchieri (National Institutes of Health [NIH]), Ulrich Siebenlist (NIH), and Ingo Schmitz (Ruhr-University Bochum, Germany), respectively.

### BMDM isolation, differentiation, and stimulation

Bone marrow was extracted from male mice aged 8–12 weeks and differentiated into BMDMs as described (Ramirez-Carrozzi et al. 2009; Bhatt et al. 2012). Media was replaced on day 4, and macrophages were treated on day 6 with 100 ng/mL lipid A (Sigma L6895) or 10 ng/mL TNF (Bio-Techne 410-MT). For MAPK inhibitor-treated samples, macrophages were pretreated with 1 μM ERK inhibitor (PD 0325901) for 1 h prior to stimulation.

### RNA-seq

For chromatin-associated RNA-seq, ∼15 million BMDMs per sample were stimulated and harvested on day 6 of differentiation. Chromatin-associated RNA and mRNA samples were prepared as described (Bhatt et al. 2012; Tong et al. 2016). Libraries were sequenced using single-end (50 bp) Illumina Hi-Seq 2000 or 3000. Reads were aligned to the NCBI37/mm9 genome using Hisat2 (Kim et al. 2015, 2019). Following alignment, SAMtools was used to compress, sort, and index files (Danecek et al. 2021). HOMER software allowed us to further process and visualize the data on the UCSC genome browser (Heinz et al. 2010). Using IGV Tools, we generated TDFs for data visualization on IGV (Robinson et al. 2011). SeqMonk was used to extract read counts from BAM files (Babraham Bioinformatics). Read counts were normalized to gene size (kilobases) and depth of sample sequencing (per million reads) to generate RPKMs.

### ChIP-seq and sequential ChIP-seq

For ChIP-seq experiments, ∼30 million (45 million for sequential ChIP-seq) BMDMs were harvested on day 6 of differentiation (two 15 cm plates per sample). RelA (Cell Signaling 8242S), p50 (Cell Signaling 13586), IκBζ (Sigma 4301779), and H3K27ac (Active Motif 39133) antibodies were used for immunoprecipitation. For sequential ChIP-seq, after the first immunoprecipitation, the protein–DNA complex was eluted from the antibody–bead complex with 10 mM DTT for 30 min at 37°C. The eluent was then diluted 20×, the second antibody was added, and the ChIP-seq protocol was continued as described (Barish et al. 2010). Reads were aligned to the NCBI37/mm9 genome using Hisat2 (Kim et al. 2015, 2019). Files were further compressed, sorted, and indexed using SAMtools (Danecek et al. 2021). IGV Tools was used to generate TDFs for data visualization on IGV (Robinson et al. 2011). Peaks were called with HOMER software using a false discovery rate of 0.01 (Heinz et al. 2010). BEDTools software allowed us to create a comprehensive list of all overlapping peaks between samples (Quinlan and Hall. 2010). We extracted raw read counts from BAM files with SeqMonk (Babraham Bioinformatics). Read counts were normalized to peak size (kilobases) and depth of sample sequencing (per million reads) to generate RPKMs. Only reproducible peaks with the described criteria were maintained for downstream analysis.

### ATAC-seq

ATAC-seq libraries were prepared with the Nextera Tn5 transposase kit (Illumina) as described (Buenrostro et al. 2015; Tong et al. 2016). Reads were mapped to the NCBI37/mm9 genome using Hisat2 (Kim et al. 2015, 2019). SAMtools was used to compress, sort, index, and remove duplicates from samples. MACS2 was used to call peaks using a false discovery rate of 0.01 (Zhang et al. 2008). To create a complete list of accessible chromatin regions in all samples, we used BEDTools (Quinlan and Hall. 2010). We extracted raw read counts from BAM files with SeqMonk (Babraham Bioinformatics). Read counts were then normalized to peak size (kilobases) and depth of sample sequencing (per million reads) to generate RPKMs.

### CRISPR/Cas9 mutagenesis

Synthetic guide RNAs (gRNAs) were designed using CRISPOR (http://crispor.tefor.net) and Massachusetts Institute of Technology's CRISPR Designer (http://crispr.mit.edu). Recombinant Cas9 (Synthego) in combination with gRNAs was electroporated into J2 transformed *Nfkb1*^−/−^ macrophages to delete exon 3 of *Nfkb2.* Single-cell clones were screened by genotyping, and loss of p52 and p50 was confirmed by immunoblot (Cell Signaling D9S3M and D4P4D) and by sequencing.

### Motif analysis

HOMER software was used to search for de novo motifs and the enrichment of position weight matrices for NF-κB from the Jaspar motif database (Heinz et al. 2010). The 200 bp surrounding the center of p50, RelA, or IκBζ ChIP-seq peaks were used in motif analyses.

### IκBζ retroviral overexpression

The IκBζ expression construct was prepared in a pMSCV vector by VectorBuilder (https://en.vectorbuilder.com). The construct was verified with DNA sequencing. Viral production was carried out by VectorBuilder and prepared to >10^7^ TU/mL. Retroviral transductions were done as described (Sanjabi et al. 2005). In brief, bone marrow was collected from mice (described above) and plated in a 6 well dish at a density of 0.5 × 10^6^cells/mL in CMG-conditioned media. On days 1–3, spin infections were performed. Cells were spun at 2500 rpm for 5 min; the supernatant was removed; and media containing the virus, 8 μg/mL polybrene, and 10 μL/mL of 1 M HEPES (pH 7.55) was incubated with cells at 2500 rpm for 1.5 h at 4°C. After spin infections, virus-containing media was removed, and CMG-conditioned media was added. On day 6, cells were stimulated and collected for either Western blotting or RNA-seq.

## Supplementary Material

Supplement 1

## Notes added in proof

The study by Zhu et al. (2024) in this issue of *Genes & Development* describes a crystal structure of IκBζ in complex with NF-κB p50 homodimers, adding support to our evidence that IκBζ can bind RelA:p50 heterodimers both in vitro and in vivo and that IκBζ binding to DNA-bound NF-κB dimers is likely to require another layer of regulation.

While our article was under review, Alpsoy et al. (2024) reported that IκBζ can associate with POU transcription factors in addition to NF-κB. In our ChIP-seq data sets, POU transcription factor motifs display modest enrichment at genomic sites bound by IκBζ, primarily at p50-independent IκBζ binding sites (data not shown), supporting a model in which interactions with POU proteins may support IκBζ binding at a subset of these p50-independent sites.
