# Supplementary material for: Selective regulation of a defined subset of inflammatory and immunoregulatory genes by an NF-κB p50–IκBζ pathway
Source: Genes Dev. 2024 Jun 1;38(11-12):536–53. doi: 10.1101/gad.351630.124 (PMC11293394; doi:10.1101/gad.351630.124)
Supplement: Supplement 1 [file Supplemental_Materials.pdf]

Supplemental Materials

Selective regulation of a defined subset of inflammatory and immunoregulatory genes by an NF-κB p50-IκBζ pathway

Allison E. Daly, George Yeh, Sofia Soltero, and Stephen T. Smale

Supplemental Figure S1

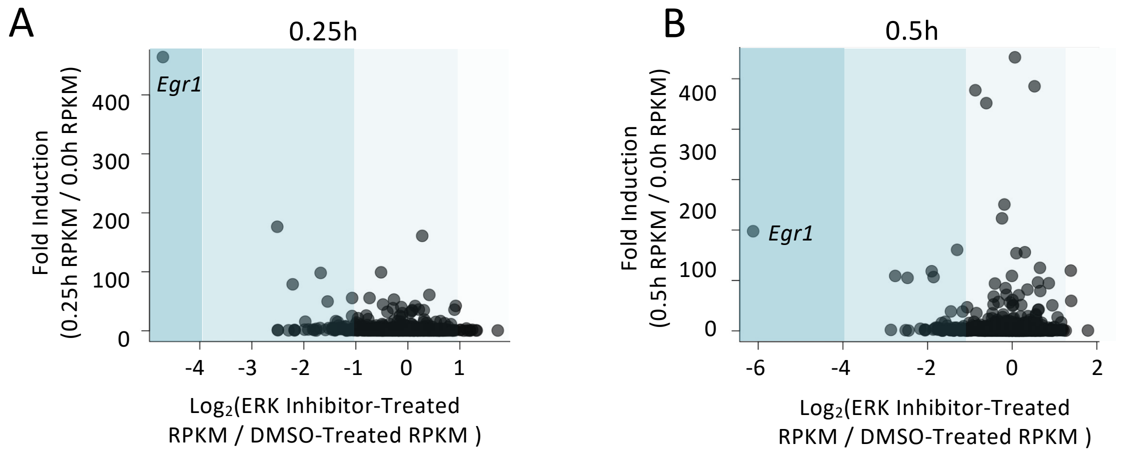

Supplemental Figure S1. Uniquely strong dependence of *Egr1* transcription on ERK signaling. BMDMs were pretreated for 1 hr with ERK inhibitor, PD-0325901 (final concentration 5 μM dissolved in DMSO) or DMSO and subsequently stimulated with lipid A. mRNA-seq was then performed with cells stimulated with lipid A for 0.25 or 0.5 hr. The scatter plot shows, on the x-axis, the log2 ratio of RPKM in ERK-inhibitor treated cells versus control cells for the 132 strongly induced primary response genes (Tong et al. 2016). The y-axis shows the fold-induction of the 132 primary response gene transcripts at the relevant time point.

Supplemental Figure S2

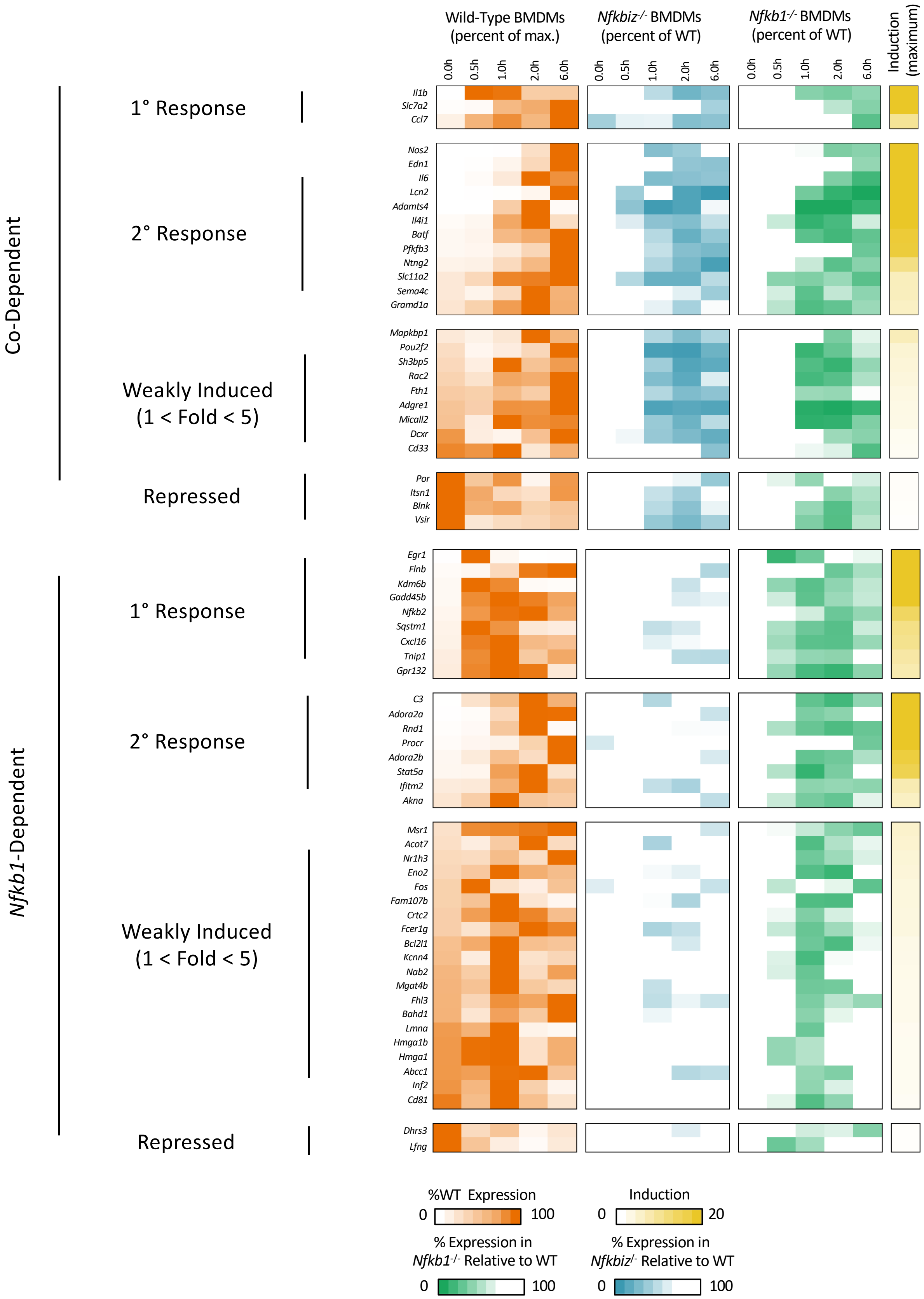

Supplemental Figure S2. Lists of *Nfkb1/Nfkbiz*-co-dependent and *Nfkb1*-dependent genes in lipid A-stimulated macrophages. Gene names and heatmaps of lipid A activation kinetics are shown (% of maximum RPKM) for the 28 *Nfkb1/Nfkbiz*-co-dependent and 39 *Nfkb1*-dependent genes. Dependence is defined as <33% chromatin-associated transcript levels at the 0.5-, 1-, 2-, or 6-hr time points in mutant cells in comparison to WT cells examined in parallel, in addition to a p-value <0.01 from two biological replicates. The genes are classified as primary response (those genes defined as primary response in Tong et al. 2016, plus additional genes induced >5-fold at any time point, with RPKM >3 at any time point, and with >33% chromatin-associated transcript levels in the presence of CHX at any time point), secondary response (those genes defined as secondary response in Tong et al. 2016 plus additional genes induced >5-fold at any time point, with RPKM >3 at any time point, and with <33% transcript level in the presence of CHX at any time point), weakly induced (those genes that not defined as primary or secondary response, but with RPKM >3 at any time point and with maximum induction in WT BMDMs of 1-5-fold), or repressed (those genes with RPKM >3 at any time point and with repression rather than induction in WT BMDMs). Heatmaps show the percent transcript level for each gene relative to the maximum transcript level observed for that gene at any time point in WT cells. The final heatmap column shows the maximum fold induction for each gene relative to the fold induction for all other genes.

Supplemental Figure S3

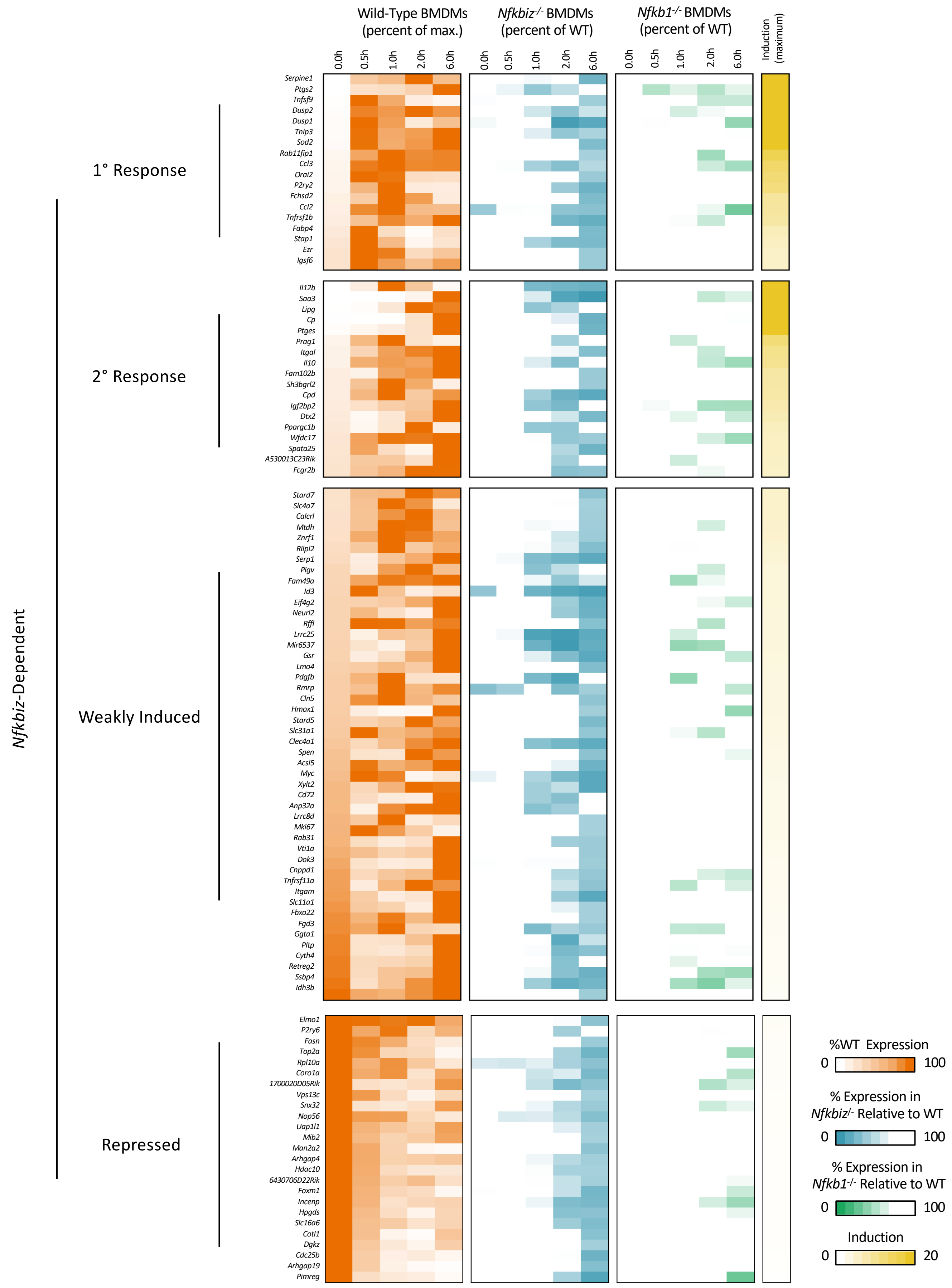

Supplemental Figure S3. A list of *Nfkbiz*-dependent genes in lipid A-stimulated macrophages. Gene names and heatmaps of lipid A activation kinetics are shown for the 108 genes with only *Nfkiz*-dependence. Definitions of dependence, classification criteria, and presentation details are the same as in Supplemental Fig. S2.

Supplemental Figure S4

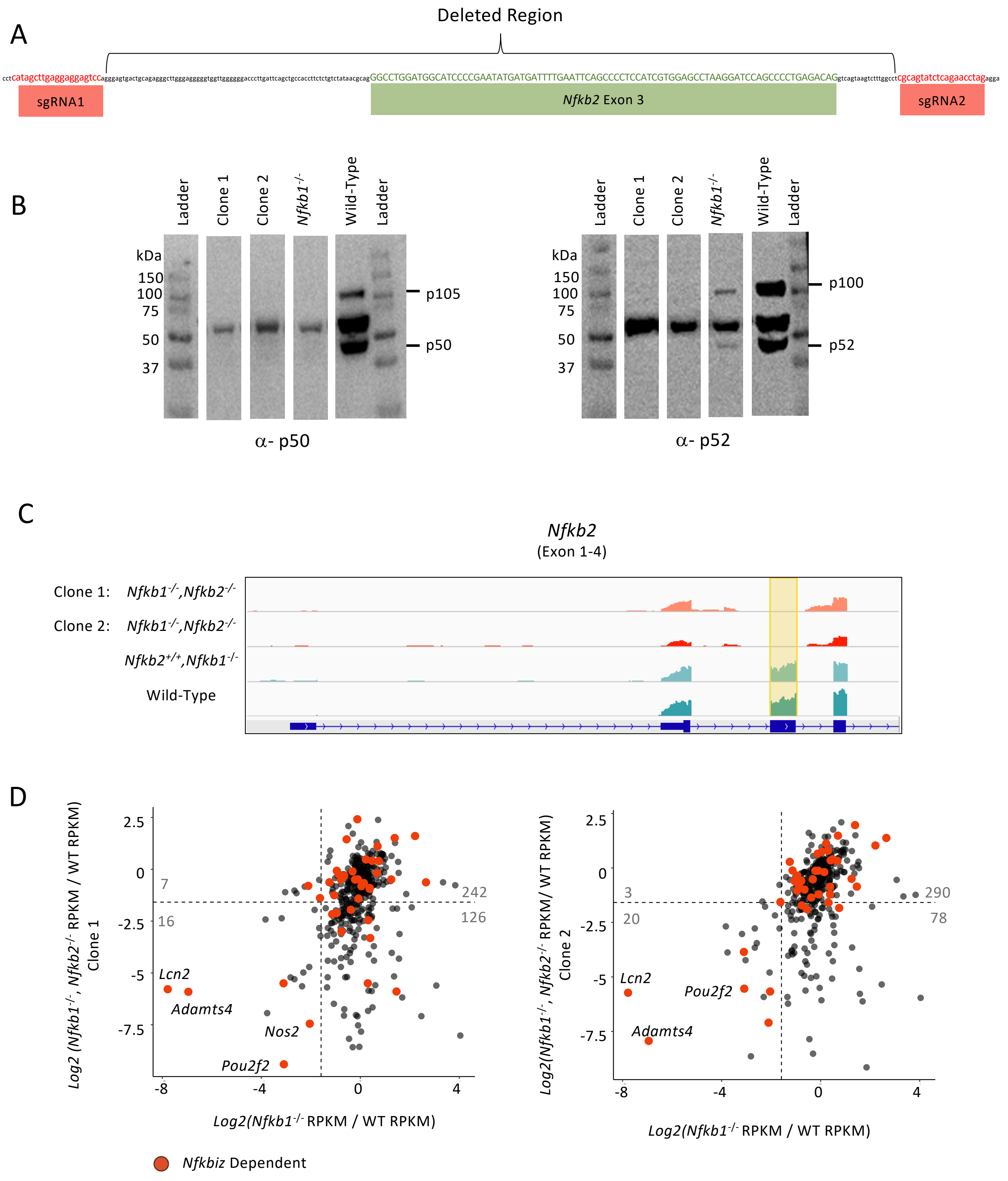

Supplemental Figure S4. Generation and analysis of Nfkb1$^{-/-}$Nfkb2$^{-/-}$ J2-transformed macrophage lines. BMDMs from *Nfkb1*$^{-/-}$ mice were transformed with the J2 retrovirus. A homozygous deletion was then introduced into the *Nfkb2* gene by CRISPR/Cas9 mutagenesis, following the protocol described in Feng et al. 2024. Two clonal *Nfkb1*$^{-/-}$*Nfkb2*$^{-/-}$ lines (clones 1 and 2) were selected and analyzed. (A) The sequence of the region surrounding *Nfkb2* exon 3 is shown, along with the two single guide RNAs (sgRNAs) used for mutagenesis. (B) Immunoblot analyses were used to confirm the absence of both the p50 (left) and p52 (right) proteins in *Nfkb1*$^{-/-}$*Nfkb2*$^{-/-}$ clones 1 and 2, with *Nfkb1*$^{-/-}$ cells and WT cells examined as controls. The p50 and p52 antibodies recognized the processed (p50 and p52) and precursor (p105 and p100) proteins translated from the two genes, as well as a non-specific protein. (C) The deletion of *Nfkb2* exon 3 was further confirmed by examination of mRNA-seq tracks from clones 1 and 2, with *Nfkb1*$^{-/-}$ cells and WT cells examined as controls. (D) Scatter plots compare the impact on mRNA levels of lipid A-induced genes in *Nfkb1*$^{-/-}$ and *Nfkb1*$^{-/-}$*Nfkb2*$^{-/-}$ macrophage lines. mRNA-seq was performed with two independent *Nfkb1*$^{-/-}$*Nfkb2*$^{-/-}$ lines, clone 1 (left) and clone 2 (right), the *Nfkb1*$^{-/-}$ parental line, and a WT J2-transformed line. The x-axes correspond to the *Nfkb1*$^{-/-}$RPKM/WT RPKM ratio for each lipid A-induced gene (induction >5-fold), and the y-axes correspond to the *Nfkb1*$^{-/-}$*Nfkb2*$^{-/-}$RPKM/WT RPKM ratio. *Nfkbiz*-dependent genes are in red. Dashed lines correspond to ratios of 0.33.
